# Effect of Patient Characteristics on Uptake of Screening Using a Mailed Human Papillomavirus Self-sampling Kit: A Secondary Analysis of a Randomized Clinical Trial

**DOI:** 10.1001/jamanetworkopen.2022.44343

**Published:** 2022-11-30

**Authors:** Rachel L. Winer, John Lin, Jasmin A. Tiro, Diana L. Miglioretti, Tara Beatty, Hongyuan Gao, Kilian Kimbel, Chris Thayer, Diana S. M. Buist

**Affiliations:** 1 Department of Epidemiology, University of Washington, Seattle; 2 Kaiser Permanente Washington Health Research Institute, Seattle; 3 Department of Population and Data Sciences, University of Texas Southwestern Medical Center, Dallas; 4 Division of Biostatistics, Department of Public Health Sciences, University of California Davis, Davis; 5 Washington Permanente Medical Group, Renton

## Abstract

**Importance:**

Mailing human papillomavirus (HPV) self-sampling kits increases cervical cancer screening participation, but effects may differ across subpopulations. Subpopulation data can inform US health care system implementation.

**Objective:**

To identify patient characteristics that modify effectiveness of a mailed kit intervention at increasing screening.

**Design, Setting, and Participants:**

This was a secondary analysis of data from the Home-Based Options to Make Cervical Cancer Screening Easy (HOME) randomized clinical trial conducted from 2014 to 2018 at Kaiser Permanente Washington. Data analysis was performed from March 2018 to May 2022. Individuals aged 30 to 64 years with female sex, health plan enrollment longer than 3 years and 5 months, a current primary care clinician, and no Papanicolaou test within the prior 3 years and 5 months were identified through electronic medical records and randomized (1:1) to the control or intervention group.

**Interventions:**

The control group received usual care Papanicolaou screening reminders and outreach. The intervention group received usual care plus an unsolicited mailed HPV self-sampling kit.

**Main Outcomes and Measures:**

Screening uptake was captured within 6 months after randomization. Baseline patient characteristics (age, race, ethnicity, travel time to clinic, income, body mass index, tobacco use, health plan enrollment duration, time since last Papanicolaou test, mammography, comorbidities, and colorectal cancer screening adherence) were extracted from the electronic medical record.

**Results:**

Of 19 734 individuals (mean [SD] age, 50.1 [9.5] years; 14 129 [71.6%] White), 9843 were randomized to the intervention group, and 9891 were randomized to the control group. Screening uptake was 26.3% (2592 of 9843 individuals) in the intervention group vs 17.4% (1719 of 9891 individuals) in the control group (relative risk [RR], 1.51; 95% CI, 1.43-1.60). Although absolute differences in uptake by group varied little by screening history, relative effects were greater with longer vs shorter time since last Papanicolaou test (no prior Papanicolaou test: RRs, 1.85-3.25; ≥10 years: RR, 2.78; 5-10 years: RRs, 1.69-1.86; <5 years: RRs 1.29-1.37). Relative effects were greater in participants overdue (RR, 2.03; 95% CI, 1.73-2.38) vs up-to-date with mammography (RR, 1.53; 95% CI, 1.41-1.67), although absolute difference was greater in the up-to-date group. Differences by age were not significant, with RRs of 1.33 to 1.48 across 5-year age groups in participants 30 to 54, vs 1.60 (95% CI, 1.40-1.82) in participants 55 to 59 and 1.77 (95% CI, 1.56-2.01) in participants 60 to 64 years. Among those mailed kits, there were differences in kit use vs in-clinic screening by age, race, plan enrollment duration, underscreening duration, and colorectal cancer screening adherence.

**Conclusions and Relevance:**

In this secondary analysis of a randomized clinical trial, clinically important improvements in screening uptake were observed for all subgroups. Differences in magnitude of intervention effect and kit use highlighted opportunities to optimize HPV self-sampling for priority groups.

**Trial Registration:**

ClinicalTrials.gov Identifier: NCT02005510

## Key Points

**Question:**

Is the effectiveness of mailed human papillomavirus self-sampling kits vs usual care (in-clinic screening reminders) on cervical cancer screening uptake modified by patient characteristics?

**Findings:**

In this secondary analysis of randomized clinical trial data, mailed kits were associated with significantly increased screening vs usual care within all subgroups of age, race and ethnicity, screening history, and other sociodemographic and health characteristics. Relative effects were greater with longer vs shorter duration of underscreening.

**Meaning:**

These findings suggest mailing kits increases screening across patient characteristics, with opportunities to optimize self-sampling for priority subgroups.

## Teaser

This randomized clinical trial evaluates the association between patient characteristics and effectiveness of a mailed human papillomavirus self-sampling kit intervention on screening uptake.

## Introduction

In the US, adherence to guideline-recommended cervical cancer screening has declined from 86% in 2005 to 77% in 2019.^1^ Increasing screening adherence is a priority,^2^ as more than 50% of the 14 000 cervical cancers diagnosed annually in the US^3^ are in individuals who are infrequently or never screened.^4,5,6,7^ Documented screening barriers include fear or embarrassment about pelvic examinations, negative prior experiences with screening, lack of a regular health care practitioner, and logistical challenges (eg, lack of time or transportation, distant proximity to a clinic, and scheduling difficulties).^4,8,9,10,11,12,13^ Additionally, screening rates vary by sociodemographics (including age, race, ethnicity, and income), body mass index (BMI), tobacco use, and adherence to other recommended cancer screening.^14,15,16,17,18,19,20,21^ Interventions are needed that effectively increase screening uptake in priority groups with low screening rates (eg, individuals who identify as American Indian/Native Alaskan, Asian, Hispanic, or Native Hawaiian/other Pacific Islander,^1,22^ have other preventive care gaps^14,19,20,23,24^ or comorbidities,^14,25,26^ are older,^14,15^ or live in rural areas^1^).

The 2018 US Preventive Services Task Force cervical cancer screening guidelines added primary human papillomavirus (HPV)–only screening as a recommended option for individuals aged 30 to 65 years.^2^ HPV testing is more sensitive than Papanicolaou testing for detecting cervical precancer,^27^ and can be performed on self-collected or clinician-collected samples with comparable sensitivity.^28,29,30^ To increase screening adherence, US health care systems may consider outreach strategies that incorporate self-sampling. International population-based trials in Europe and Australia have consistently demonstrated that mailing self-sampling kits increases screening participation compared with clinic-based screening invitations.^28^ Accelerated by the COVID-19 pandemic, multiple countries have incorporated HPV self-sampling options into their cervical cancer screening programs.^31^

Home-Based Options to Make Cervical Cancer Screening Easy (HOME)^32^ was the first trial to evaluate pragmatic effectiveness of a programmatic mailed HPV self-sampling kit outreach strategy in a US health care system. Mailing kits to underscreened individuals increased screening compared with usual care reminders and outreach.^33^ However, nearly three-quarters of individuals in the intervention group did not screen, and the increase in screening associated with the intervention was in the lower range of estimates from international trials,^28^ highlighting improvement opportunities. Limited data from international trials suggest potential differences in population subgroups that are best reached through HPV self-sampling.^28,34,35,36,37,38,39,40^ To inform US health care system implementation, the objective of this secondary data analysis was to identify patient characteristics that modify the mailed kit intervention’s effectiveness at increasing screening uptake.

## Methods

Within HOME,^32,33^ we evaluated whether patient characteristics modified effectiveness of a mailed HPV kit intervention at increasing screening uptake. HOME was approved by Kaiser Permanente Washington (KPWA) and University of Washington institutional review boards. Design details were described previously.^32^ Consolidated Standards of Reporting Trials (CONSORT) reporting guidelines were followed (Figure). The protocol is available in the eAppendix in Supplement 1.

**Figure.**
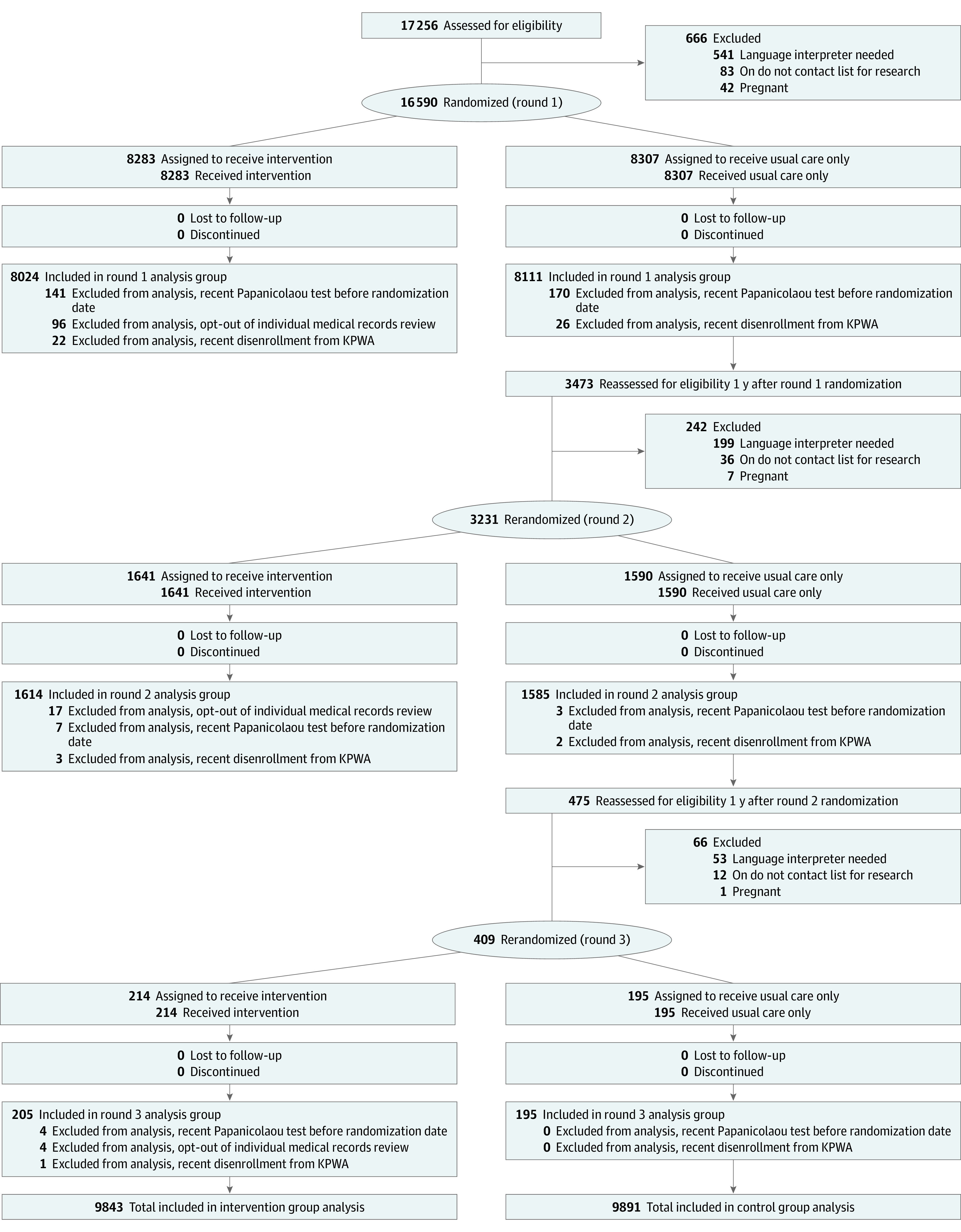
Flow Diagram of Trial Participation KPWA indicates Kaiser Permanente Washington.

Briefly, HOME evaluated whether mailing kits to individuals overdue for cervical cancer screening increased screening uptake, detection, and treatment of cervical neoplasia compared with usual care. From February 25, 2014, to August 29, 2016, we used electronic medical record (EMR) data to identify individuals with female sex, aged 30 to 64 years, with an intact uterus, continuously enrolled at KPWA for 3 years and 5 months or more, and with no Papanicoloau test within 3 years and 5 months. Individuals were excluded if they were on a do-not-contact list for research, were pregnant, or had an “interpreter needed” flag. All eligible individuals were enrolled under a full waiver of consent 5 months after receiving an annual preventive services reminder letter indicating they were due or overdue for screening. Patients were randomly allocated 1:1 to the intervention or control group. To meet sample size targets, control group participants were reassessed for eligibility and rerandomization 1 year after randomization. Control group participants received usual care outreach to attend Papanicoloau screening (described previously)^32^ and were not contacted by the study team. Intervention group participants received usual care plus a mailed HPV self-sampling kit with prepaid return mailer. Kit mailings included a research information sheet with a telephone number to opt out of having individual-level medical record data used for research. Letters recommended routine in-clinic Papanicolaou screening, regardless of kit use, because HPV testing on self-collected samples is not standard care in the US. HPV results were documented in the EMR, and participants’ primary care teams were responsible for communicating abnormal results and scheduling follow-up.

Screening uptake was a prespecified secondary outcome in the original randomized clinical trial captured 6 or fewer months after randomization from EMR data and defined as follows: (1) in-clinic screening; (2) self-sampling HPV 16/18-positive; (3) self-sampling HPV-negative; or (4) self-sampling HPV-positive for non-HPV 16/18 types only or unsatisfactory, followed by in-clinic screening. Patient characteristics at randomization were also derived from EMR data: age, race and ethnicity (self-reported; categories were American Indian/Alaska Native, Asian, Black or African American, Native Hawaiian or other Pacific Islander, White, other [those selecting other were further able to enter a free text description; institutional review board approval did not allow individual-level data for all participants, so to maintain consistency across all participants in categorization of race, no free text entries were reviewed or coded], and unknown), health plan enrollment duration, time since last Papanicolaou test stratified by health plan enrollment duration, US Census block median household income, travel time to primary care clinic,^41^ BMI, tobacco use, Charlson Comorbidity Index,^42^ and Healthcare Effectiveness Data and Information Set–defined adherence to guideline-recommended mammography^43^ (restricted to those aged 52-64 years) and colorectal cancer (CRC) screening^44^ (restricted to those aged 51-64 years). Given documented racial and ethnic disparities in cervical cancer screening in the US,^22^ race and ethnicity were analyzed to identify potential differences in intervention effectiveness across groups.

### Statistical Analysis

As prespecified in our analysis plan,^32^ we used intention-to-treat (ITT) log-binomial regression to estimate risk differences and relative risks (RRs) with 95% CIs for associations between randomization group and screening, and evaluate patient-characteristic-by-randomization group interactions. CONSORT guidelines recommend reporting both absolute and relative effect sizes as complementary measures.^45^ Relative and absolute intervention effect sizes may differ across patient subgroups with varying baseline outcome rates, and absolute differences may be particularly relevant for informing health care system decision-making. Each observation was treated separately in analysis according to group assignment, as per the ITT, with robust variance estimates used to account for within-participant correlation due to rerandomized participants contributing more than 1 observation period. Denominators for each group included all participants randomized, minus the small number who opted out of medical record review or were identified after randomization as ineligible. Statistical significance was defined as a 2-sided *P* < .05.

In posthoc analysis, we estimated screening uptake within the intervention group by modality (initiating screening by kit return vs in-clinic), and tested for patient-characteristic-by-modality interactions using χ^2^ tests. We also tested for patient-characteristic-by-randomization group interactions for in-clinic screening (screening in-clinic in the intervention group vs screening in-clinic in the control group) using log-binomial regression and robust variance estimates. Analyses were conducted after the trial ended between March 2018 to May 2022 using SAS statistical software version 9.4 (SAS Institute).

## Results

The total number of randomized participants was 20 230 (Figure). We retroactively excluded 379 who screened in-clinic before randomization or disenrolled from KPWA close to randomization. For analyses described here, 117 (1.2%) intervention group participants who opted out of individual-level medical record review (with unavailable patient characteristic data) were also excluded, leaving 9843 in the intervention group and 9891 in the control group. The mean (SD) age of these 19 734 individuals was 50.1 (9.5) years. A total of 14 129 (71.6%) individuals were White. Baseline characteristics between groups were similar.^33^

### Prespecified Secondary Outcomes

Screening uptake was higher in the intervention group (2592 of 9843 individuals [26.3%]) vs the control group (1719 of 9891 individuals [17.4%]), corresponding to a RR of 1.51 (95% CI, 1.43-1.60) and absolute screening uptake difference of 8.9% (95% CI, 7.8%-10.0%). Across patient characteristics, nearly all stratum-specific RR and absolute risk differences showed clinically relevant and statistically significant increases in screening between groups (Table 1). The relative effect was modified by screening history, with greater RRs for longer vs shorter time since last Papanicolaou test (no prior Papanicolaou test, RRs, 1.85-3.25; ≥10 years prior, RR, 2.78; 5-10 years, RRs, 1.69-1.86; <5 years, RRs, 1.29-1.37) (*P* for interaction ≤ .005 for all comparisons). Relative differences in intervention effect size are impacted by screening uptake in the control group, which ranged from 2.7% to 10.3% in participants with no prior screening to 27.0% to 29.0% in those with less than 5 years since last screen. Conversely, absolute differences varied little by screening history (no prior Papanicolaou test, absolute differences, 6.1%-8.7%; ≥10 years prior, 8.1%; 5-10 years, 9.0%-11.0%; <5 years, 7.8%-10.6%). The relative effect of the intervention was also greater in participants overdue (RR, 2.03; 95% CI, 1.73-2.38) vs up-to-date (RR, 1.53; 95% CI, 1.41-1.67; *P* = .002) for mammography, although the absolute difference was greater in the up-to-date (13.6%) vs not up-to-date (7.9%) group. There were no significant differences in relative effect of the intervention by age, with RRs ranging from 1.33 to 1.48 across 5-year age groups in participants aged 30 to 54 years, vs RRs of 1.60 (95% CI, 1.40-1.82) in participants aged 55 to 59 years and 1.77 (95% CI, 1.56-2.01) in participants aged 60 to 64 years. No other significant patient-characteristic-by-randomization group interactions were found.

**Table 1.** Effectiveness of a Mailed Human Papillomavirus Kit Intervention vs Usual Care for Increasing Screening Uptake in Underscreened Individuals in a US Health Care System, Stratified by Select Patient Characteristics

Characteristic	Participants, No./total No. (%)		Absolute difference between groups, % (95% CI)^b^	Intervention vs control, RR (95% CI)^c^	*P* value for characteristic-by-group interaction
	Intervention (n = 9843^a^)	Control (n = 9891)			
Age, y					
30-34	206/808 (25.5)	144/794 (18.1)	7.4 (3.3-11.4)	1.41 (1.16-1.70)	.08
35-39	259/932 (27.8)	191/915 (20.9)	6.9 (3.0-10.8)	1.33 (1.13-1.57)	
40-44	338/1194 (28.3)	240/1185 (20.3)	8.1 (4.6-11.5)	1.40 (1.21-1.61)	
45-49	350/1380 (25.4)	239/1374 (17.4)	8.0 (4.9-11.0)	1.46 (1.26-1.69)	
50-54	433/1682 (25.7)	297/1707 (17.4)	8.3 (5.6-11.1)	1.48 (1.30-1.69)	
55-59	478/1938 (24.7)	300/1943 (15.4)	9.2 (6.7-11.7)	1.60 (1.40-1.82)	
60-64	528/1909 (27.7)	308/1973 (15.6)	12.0 (9.5-14.6)	1.77 (1.56-2.01)	
Race^d^					
American Indian/Alaska Native	28/147 (19.0)	15/145 (10.3)	8.7 (0.6-16.8)	1.84 (1.03-3.30)	.51
Asian	247/893 (27.7)	171/880 (19.4)	8.2 (4.3-12.1)	1.42 (1.20-1.69)	
Black or African American	119/438 (27.2)	71/431 (16.5)	10.7 (5.3-16.1)	1.65 (1.27-2.14)	
More than 1 race	74/285 (26.0)	49/283 (17.3)	8.7 (1.9-15.4)	1.50 (1.08-2.07)	
Native Hawaiian or other Pacific Islander	32/151 (21.2)	22/139 (15.8)	5.4 (−3.6-14.3)	1.34 (0.82-2.20)	
Other	63/250 (25.2)	53/235 (22.6)	2.6 (−5.0-10.3)	1.12 (0.81-1.54)	
White	1975/7018 (28.1)	1289/7111 (18.1)	10.0 (8.6-11.4)	1.55 (1.46-1.65)	
Unknown	54/661 (8.2)	49/667 (7.3)	NA	NA	
Ethnicity^d^					
Hispanic	128/486 (26.3)	96/480 (20.0)	6.3 (1.0-11.6)	1.32 (1.04-1.66)	.22
Non-Hispanic	2403/8710 (27.6)	1577/8761 (18)	9.6 (8.4-10.8)	1.53 (1.45-1.62)	
Unknown	61/647 (9.4)	46/650 (7.1)	NA	NA	
Length of health plan enrollment, y					
3.4 to <5	556/2230 (24.9)	359/2240 (16.0)	8.9 (6.6-11.3)	1.56 (1.38-1.75)	0.78
5 to <10	779/3115 (25.0)	516/3045 (16.9)	8.1 (6.1-10.1)	1.48 (1.34-1.63)	
≥10	1257/4498 (27.9)	844/4606 (18.3)	9.6 (7.9-11.3)	1.53 (1.41-1.65)	
Time since last Papanicolaou test (by length of health plan enrollment)					
Enrolled 3.4 to <5 y					
>3.4 to <5	266/704 (37.8)	202/710 (28.5)	9.3 (4.5-14.2)	1.33 (1.14-1.54)	.005
No Papanicolaou test	290/1526 (19.0)	157/1530 (10.3)	8.7 (6.3-11.2)	1.85 (1.55-2.22)	
Enrolled 5 to <10 y					
>3.4 to <5	530/1519 (34.9)	397/1468 (27.0)	7.8 (4.6-11.1)	1.29 (1.16-1.44)	<.001
5 to <10	119/540 (22.0)	66/507 (13.0)	9.0 (4.5-13.6)	1.69 (1.29-2.23)	
No Papanicolaou test	130/1056 (12.3)	53/1070 (5.0)	7.4 (5.0-9.7)	2.49 (1.83-3.38)	
Enrolled ≥10 y					
>3.4 to <5	864/2186 (39.5)	652/2252 (29.0)	10.6 (7.8-13.4)	1.37 (1.26-1.48)	<.001
5 to <10	272/1143 (23.8)	151/1182 (12.8)	11.0 (7.9-14.1)	1.86 (1.55-2.23)	
≥10	60/475 (12.6)	23/506 (4.5)	8.1 (4.6-11.6)	2.78 (1.75-4.41)	
No Papanicolaou test	61/694 (8.8)	18/666 (2.7)	6.1 (3.6-8.5)	3.25 (1.94 − 5.45)	
US Census block, median household income, $US^e^					
<25 000	38/140 (27.1)	17/125 (13.6)	13.5 (4.1-23.0)	2.00 (1.19-3.34)	.56
25 000-49 999	506/2107 (24.0)	326/2115 (15.4)	8.6 (6.2-11.0)	1.56 (1.37-1.77)	
50 000-74 999	901/3448 (26.1)	568/3439 (16.5)	9.6 (7.7-11.5)	1.58 (1.44-1.74)	
75 000-99 999	670/2405 (27.9)	477/2483 (19.2)	8.7 (6.3-11.0)	1.45 (1.31-1.61)	
≥100 000	314/1025 (30.6)	212/1013 (20.9)	9.7 (5.9-13.5)	1.46 (1.26-1.70)	
Unknown	163/718 (22.7)	119/716 (16.6)	NA	NA	
Travel time from home to primary care clinic, min^f^					
< 10	863/3254 (26.5)	544/3236 (16.8)	9.7 (7.7-11.7)	1.58 (1.43-1.74)	.53
10 to <20	1061/4086 (26.0)	722/4048 (17.8)	8.2 (6.3-9.9)	1.46 (1.34-1.58)	
20 to <30	398/1407 (28.3)	255/1415 (18.0)	10.3 (7.2-13.4)	1.57 (1.37-1.80)	
≥30	249/1004 (24.8)	186/1072 (17.4)	7.4 (3.9-11.0)	1.43 (1.21-1.69)	
Unknown	21/92 (22.8)	12/120 (10.0)	NA	NA	
Body mass index^g^					
<18.5	28/109 (25.7)	19/98 (19.4)	6.3 (−5.0-17.6)	1.32 (0.79-2.21)	.63
18.5 to 24.9	729/2238 (32.6)	512/2248 (22.8)	9.8 (7.2-12.4)	1.43 (1.30-1.58)	
25 to 29.9	676/2168 (31.2)	467/2220 (21.0)	10.1 (7.6-12.7)	1.48 (1.34-1.64)	
30 to 34.9	443/1549 (28.6)	278/1603 (17.3)	11.3 (8.3-14.2)	1.65 (1.44-1.88)	
35 to 39.9	297/1119 (26.5)	200/1080 (18.5)	8.0 (4.5-11.5)	1.43 (1.22-1.68)	
≥40	290/1248 (23.2)	190/1184 (16)	7.2 (4.0-10.4)	1.45 (1.23-1.71)	
Unknown	129/1412 (9.1)	53/1458 (3.6)	NA	NA	
Tobacco use					
Current	236/1276 (18.5)	159/1290 (12.3)	6.2 (3.4-9.0)	1.50 (1.25-1.81)	.77
Former	625/2041 (30.6)	400/2020 (19.8)	10.8 (8.2-13.5)	1.55 (1.39-1.73)	
Never	1613/5237 (30.8)	1092/5232 (20.9)	9.9 (8.3-11.6)	1.48 (1.38-1.58)	
Unknown	118/1289 (9.2)	68/1349 (5.0)	NA	NA	
Charlson Comorbidity Index^h^					
0	2122/7967 (26.6)	1386/8052 (17.2)	9.4 (8.2-10.7)	1.55 (1.46-1.64)	.47
1	283/1087 (26.0)	208/1128 (18.4)	7.6 (4.1-11.1)	1.41 (1.20-1.66)	
2	106/432 (24.5)	72/385 (18.7)	5.8 (0.2-11.5)	1.31 (1.01-1.71)	
≥3	81/357 (22.7)	53/326 (16.3)	6.4 (0.6-12.3)	1.40 (1.02-1.90)	
Adherent to guideline-recommended breast cancer screening^i^					
No	396/2551 (15.5)	198/2587 (7.7)	7.9 (6.1-9.6)	2.03 (1.73-2.38)	.002
Yes	868/2221 (39.1)	586/2301 (25.5)	13.6 (10.9-16.3)	1.53 (1.41-1.67)	
Unknown	19/158 (12.0)	14/155 (9.0)	NA	NA	
Adherent to guideline-recommended colorectal cancer screening^j^					
No	527/3106 (17.0)	329/3240 (10.2)	6.8 (5.1-8.5)	1.67 (1.47-1.90)	.39
Yes	816/1990 (41.0)	519/1974 (26.3)	14.7 (11.8-17.6)	1.56 (1.42-1.71)	
Unknown	12/153 (7.8)	11/135 (8.1)	NA	NA	

Abbreviation: NA, not applicable.

^a^ Patient characteristics are not available for 117 (1.2%) participants in the intervention group who opted out of electronic medical record review.

^b^ The 95% CIs are from log-binomial regression models with identity link function.

^c^ The 95% CIs are from log-binomial regression models with log link function.

^d^ Race and ethnicity from electronic medical record data per patient self-report at usual care patient registration via preset multiselect categorical options, with “other” allowing free text entry. The study variable was programmatically categorized into the displayed categories by coding any multiple selections as “more than 1 race.” Manual coding of the "other" category was precluded because institutional review board approval only allowed for individual-level data for the control arm and intervention arm kit returners.

^e^ Individual household income data were not available in the EHR; as a proxy, we used median household income calculated at women’s US Census block.

^f^ Travel time to primary care clinic was generated with Network Analyst (ArcInfo v 9.1) using geographic centroids of US Census blocks and geocoded street address using women’s home addresses.^41^

^g^ Body mass index is calculated as weight in kilograms divided by height in meters squared.

^h^ Generated from an additive index of comorbid conditions.^42^

^i^ Restricted to participants 52 to 64 years old; adherence is based on Healthcare Effectiveness Data and Information Set (HEDIS) definition.^43^

^j^ Restricted to participants 51 to 64 years old; adherence is based on HEDIS definition.^44^

### Post hoc Analyses

Within the intervention group, 12.2% of participants (1201 of 9843 participants) initiated screening by kit and 14.4% (1419 of 9843 participants) did so in-clinic, corresponding to 45.8% (1201 of 2620 screenings) of intervention group screenings initiated by kit and 54.2% (1419 of 2620 screenings) initiated in-clinic. Screening modality differed by age, race, plan enrollment duration, time since last Papanicolaou test, and CRC screening adherence; all characteristic-by-modality interactions were significant, as shown in Table 2. Among screened participants, the proportion using kits was higher in older vs younger age groups. More than half of screened participants aged 50 to 64 years used a kit (ranging from 219 of 436-290 of 534 [50.2%-54.3%] across 5-year age groups), compared with slightly more than one-third of screened participants aged 30 to 44 years (70 of 210-99 of 263 [33.3%-37.6%]). Additionally, differences across racial groups were seen in proportion of screened participants who used kits (12 of 28 [42.9%] in American Indian/Alaska Native, 99 of 247 [40.1%] in Asian, 47 of 120 [39.2%] in Black/African American, 8 of 33 [24.2%] in Native Hawaiian/other Pacific Islander, and 950 of 1996 [47.6%] in White participants). The proportion using kits was similar among Hispanic (59 of 132 [44.7%]) and non-Hispanic (1116 of 2425 [46.0%]) participants. Screening by kit was also more common among participants enrolled in KPWA for 10 or more years (622 of 1267 [49.1%] of screened participants) vs 3.4 to less than 5 years (254 of 562 [45.2%]) or 5 to less than 10 (325 of 791 [41.1%]) years. There were also differences by time since last Papanicolaou test. The proportion screened by kit was greatest for individuals with longer time since last screen (≥10 years; 42 of 62 [67.7%]) and with no prior screening documented (150 of 293 to 49 of 62 [51.2%-79.0%]), intermediate among those with 5 to less than 10 years since last screen (55 of 123 to 157 of 276 [44.7%-56.9%]), and lowest among those with last screen less than 5 years prior (195 of 534 to 374 of 867 [36.5%-43.1%]). The proportion of screened participants using kits was higher among participants up-to-date vs not up-to-date with CRC screening (452 of 824 [54.9%] vs 262 of 532 [49.2%]), whereas the reverse was observed among participants up-to-date vs not up-to-date with mammography (452 of 874 [51.7%] vs 230 of 403 [57.1%]).

**Table 2.** Screening Uptake by Modality in the Intervention and Control Groups, Stratified by Select Patient Characteristics

Characteristic	Intervention group (n = 9843^a^)				Control group (n = 9891)	
	Participants, No./total No. (%)		Screened participants who returned an HPV kit, No./total No. screened (%)	*P* value for characteristic-by-screening modality (kit vs in-clinic) interaction within the intervention group^b^	Participants, No./total No. (%)	*P* value for characteristic-by-randomization group interaction (intervention group in-clinic screening vs control group in-clinic screening)^c^
	Screened with HPV kit	Screened in-clinic				
Age, y						
30-34	70/808 (8.7)	140/808 (17.3)	70/210 (33.3)	<.001	144/794 (18.1)	.31
35-39	99/932 (10.6)	164/932 (17.6)	99/263 (37.6)		191/915 (20.9)	
40-44	116/1194 (9.7)	226/1194 (18.9)	116/342 (33.9)		240/1185 (20.3)	
45-49	150/1380 (10.9)	203/1380 (14.7)	150/353 (42.5)		239/1374 (17.4)	
50-54	219/1682 (13.0)	217/1682 (12.9)	219/436 (50.2)		297/1707 (17.4)	
55-59	257/1938 (13.3)	225/1938 (11.6)	257/482 (53.3)		300/1943 (15.4)	
60-64	290/1909 (15.2)	244/1909 (12.8)	290/534 (54.3)		308/1973 (15.6)	
Race^d^						
American Indian/Alaska Native	12/147 (8.2)	16/147 (10.9)	12/28 (42.9)	.03	15/145 (10.3)	.47
Asian	99/893 (11.1)	148/893 (16.6)	99/247 (40.1)		171/880 (19.4)	
Black or African American	47/438 (10.7)	73/438 (16.7)	47/120 (39.2)		71/431 (16.5)	
More than 1 race	33/285 (11.6)	42/285 (14.7)	33/75 (44.0)		49/283 (17.3)	
Native Hawaiian or Other Pacific Islander	8/151 (5.3)	25/151 (16.6)	8/33 (24.2)		22/139 (15.8)	
Other	31/250 (12.4)	34/250 (13.6)	31/65 (47.7)		53/235 (22.6)	
White	950/7018 (13.5)	1046/7018 (14.9)	950/1996 (47.6)		1289/7111 (18.1)	
Unknown	21/661 (3.2)	35/661 (5.3)	21/56 (37.5)		49/667 (7.3)	
Ethnicity^d^						
Hispanic	59/486 (12.1)	73/486 (15.0)	59/132 (44.7)	.77	96/480 (20.0)	.46
Non-Hispanic	1116/8710 (12.8)	1309/8710 (15.0)	1116/2425 (46.0)		1577/8761 (18.0)	
Unknown	26/647 (4.0)	37/647 (5.7)	26/63 (41.3)		46/650 (7.1)	
Length of health plan enrollment, y						
3.4 to <5	254/2230 (11.4)	308/2230 (13.8)	254/562 (45.2)	.002	359/2240 (16.0)	.23
5 to <10	325/3115 (10.4)	466/3115 (15.0)	325/791 (41.1)		516/3045 (16.9)	
≥10	622/4498 (13.8)	645/4498 (14.3)	622/1267 (49.1)		844/4606 (18.3)	
Enrolled 3.4 to <5 y						
>3.4 to <5	104/704 (14.8)	165/704 (23.4)	104/269 (38.7)	.003	202/710 (28.5)	.47
No Papanicolaou test	150/1526 (9.8)	143/1526 (9.4)	150/293 (51.2)		157/1530 (10.3)	
Enrolled 5 to <10 y						
>3.4 to <5	195/1519 (12.8)	339/1519 (22.3)	195/534 (36.5)	<.001	397/1468 (27.0)	.22
5 to <10	55/540 (10.2)	68/540 (12.6)	55/123 (44.7)		66/507 (13.0)	
No Papanicolaou test	75/1056 (7.1)	59/1056 (5.6)	75/134 (56.0)		53/1070 (5.0)	
Enrolled ≥10 y						
>3.4 to <5	374/2186 (17.1)	493/2186 (22.6)	374/867 (43.1)	<.001	652/2252 (29.0)	.91
5 to <10	157/1143 (13.7)	119/1143 (10.4)	157/276 (56.9)		151/1182 (12.8)	
≥10	42/475 (8.8)	20/475 (4.2)	42/62 (67.7)		23/506 (4.5)	
No Papanicolaou test	49/694 (7.1)	13/694 (1.9)	49/62 (79.0)		18/666 (2.7)	
US Census block, median household Income, $US^e^						
<25 000	20/140 (14.3)	18/140 (12.9)	20/38 (52.6)	.46	17/125 (13.6)	.35
25 000-49 999	226/2107 (10.7)	286/2107 (13.6)	226/512 (44.1)		326/2115 (15.4)	
50 000-74 999	412/3448 (11.9)	503/3448 (14.6)	412/915 (45)		568/3439 (16.5)	
75 000-99 999	326/2405 (13.6)	347/2405 (14.4)	326/673 (48.4)		477/2483 (19.2)	
≥100 000	140/1025 (13.7)	175/1025 (17.1)	140/315 (44.4)		212/1013 (20.9)	
Unknown	77/718 (10.7)	90/718 (12.5)	77/167 (46.1)		119/716 (16.6)	
Travel time from home to primary care clinic, min^f^						
< 10	395/3254 (12.1)	477/3254 (14.7)	395/872 (45.3)	.54	544/3236 (16.8)	.30
10-<20	506/4086 (12.4)	563/4086 (13.8)	506/1069 (47.3)		722/4048 (17.8)	
20-<30	174/1407 (12.4)	228/1407 (16.2)	174/402 (43.3)		255/1415 (18.0)	
≥30	116/1004 (11.6)	140/1004 (13.9)	116/256 (45.3)		186/1072 (17.4)	
Unknown	10/92 (10.9)	11/92 (12.0)	10/21 (47.6)		12/120 (10.0)	
Body mass index^g^						
<18.5	13/109 (11.9)	15/109 (13.8)	13/28 (46.4)	.44	19/98 (19.4)	.32
18.5 to 24.9	322/2238 (14.4)	411/2238 (18.4)	322/733 (43.9)		512/2248 (22.8)	
25 to 29.9	318/2168 (14.7)	365/2168 (16.8)	318/683 (46.6)		467/2220 (21.0)	
30 to 34.9	186/1549 (12.0)	261/1549 (16.8)	186/447 (41.6)		278/1603 (17.3)	
35 to 39.9	147/1119 (13.1)	155/1119 (13.9)	147/302 (48.7)		200/1080 (18.5)	
≥40	132/1248 (10.6)	164/1248 (13.1)	132/296 (44.6)		190/1184 (16.0)	
Unknown	83/1412 (5.9)	48/1412 (3.4)	83/131 (63.4)		53/1458 (3.6)	
Tobacco use						
Current	112/1276 (8.8)	128/1276 (10.0)	112/240 (46.7)	.66	159/1290 (12.3)	.94
Former	292/2041 (14.3)	342/2041 (16.8)	292/634 (46.1)		400/2020 (19.8)	
Never	721/5237 (13.8)	905/5237 (17.3)	721/1626 (44.3)		1092/5232 (20.9)	
Unknown	76/1289 (5.9)	44/1289 (3.4)	76/120 (63.3)		68/1349 (5.0)	
Charlson Comorbidity Index^h^						
0	962/7967 (12.1)	1179/7967 (14.8)	962/2141 (44.9)	.24	1386/8052 (17.2)	.14
1	141/1087 (13.0)	147/1087 (13.5)	141/288 (49.0)		208/1128 (18.4)	
2	57/432 (13.2)	53/432 (12.3)	57/110 (51.8)		72/385 (18.7)	
≥3	41/357 (11.5)	40/357 (11.2)	41/81 (50.6)		53/326 (16.3)	
Adherent to guideline-recommended breast cancer screening^i^						
Participants, No.	4930	4930			5043	
No	230/2551 (9.0)	173/2551 (6.8)	230/403 (57.1)	.07	198/2587 (7.7)	.003
Yes	452/2221 (20.4)	422/2221 (19.0)	452/874 (51.7)		586/2301 (25.5)	
Unknown	12/158 (7.6)	7/158 (4.4)	12/19 (63.2)		14/155 (9.0)	
Adherent to guideline-recommended colorectal cancer screening^j^						
Participants, No.	5249	5249			5349	
No	262/3106 (8.4)	270/3106 (8.7)	262/532 (49.2)	.04	329/3240 (10.2)	.39
Yes	452/1990 (22.7)	372/1990 (18.7)	452/824 (54.9)		519/1974 (26.3)	
Unknown	7/153 (4.6)	5/153 (3.3)	7/12 (58.3)		11/135 (8.1)	

^a^ Patient characteristics are not available for 117 participants in the intervention group who opted out of electronic medical record review.

^b^ *P* values are from χ^2^ tests comparing kit vs Papanicolaou within the intervention group.

^c^ *P* values are from log-binomial regression models comparing intervention group Papanicolaou vs control group Papanicolaou.

^d^ Race and ethnicity from electronic medical record data per patient self-report at usual care patient registration via preset multiselect categorical options with "other" allowing free text entry. The study variable was programmatically categorized into the displayed categories by coding any multiple selections as “more than 1 race.” Manual coding of the “other” category was precluded because institutional review board approval only allowed for individual-level data for the control arm and intervention arm kit returners.

^e^ Individual household income data were not available in the electronic health record; as a proxy, we used median household income calculated at women’s US Census block.

^f^ Travel time to primary care clinic was generated with Network Analyst (ArcInfo v 9.1) using geographic centroids of US Census blocks and geocoded street address using women’s home addresses.^41^

^g^ Body mass index is calculated as weight in kilograms divided by height in meters squared.

^h^ Generated from an additive index of comorbid conditions.^42^

^i^ Restricted to participants 52 to 64 years old; adherence is based on Healthcare Effectiveness Data and Information Set (HEDIS) definition.^43^

^j^ Restricted to participants 51 to 64 years old; adherence is based on HEDIS definition.^44^

Across most characteristics, in-clinic screening in the control group was higher than in-clinic screening in the intervention group (Table 2). The between-group difference for in-clinic screening was greater among those up-to-date vs not with mammography (absolute difference, 6.5% vs 0.9%; *P* = .003). No other significant characteristic-by-randomization group interactions were seen for in-clinic screening in the intervention vs control group.

## Discussion

In this secondary analysis of randomized clinical trial data, mailing unsolicited HPV kits to underscreened individuals increased screening compared with usual care within all subgroups evaluated. Cervical cancer screening history and mammography adherence were the only patient characteristics that modified the intervention effect. Overall, the majority of intervention group participants remained unscreened. Within the intervention group, the proportion of screened participants choosing kits vs in-clinic screening differed by several patient characteristics, highlighting opportunities to optimize self-sampling for priority groups.

Although screening uptake in the mailed kit group (26.3%) was similar to screening uptake in underscreened individuals in a meta-analysis of international trials (25%),^28^ the proportion of HOME intervention group participants who screened by kit vs in-clinic was smaller, and differences in screening between groups was in the lower range of estimates from international trials.^28^ Thus, we sought to identify patient characteristics that modified the intervention effect or were differentially associated with kit uptake. The intervention effect was modified by cervical cancer screening history. Relative increases in screening were greater with longer vs shorter time since last screen, and greatest in individuals with no prior screening. In the intervention group, choice of screening modality varied by screening history. Individuals without prior screening were most likely to screen by kit vs in-clinic, followed by those with longer vs shorter time since last screening. International trials (with population-based registries to document screening history) demonstrated that mailing kits to long-term nonattenders increases screening participation,^36,46,47,48,49^ and some^49,50,51^ but not all^34,36,48^ showed larger relative effects with longer vs shorter nonattendance duration. However, relative differences in intervention effect size are impacted by screening uptake in the control group, which in HOME ranged from 3% to 10% in participants with no prior screening to 27% to 29% in those overdue by less than 2 years. Of note, absolute differences in screening by randomization group varied little by screening history. It is unsurprising that we observed differences in underlying screening uptake and modality in the US vs other international settings, because (1) the US lacks centralized national cancer screening programs, and (2) there are different implementation challenges and opportunities for HPV self-sampling.

Cervical, breast, and CRC underscreening are associated and share common correlates.^14,19,20,23,24^ We found that mailed kits were effective at increasing cervical screening uptake in difficult-to-reach individuals who were also overdue for other recommended cancer screening. Relative intervention effect was greater in those overdue vs up-to-date with mammography, and there was a similar but nonsignificant difference by CRC screening adherence. Importantly, however, absolute differences in cervical screening uptake between groups were smaller for participants overdue for these other cancer screenings vs up-to-date. Results highlight the need for multipronged interventions to reduce cervical cancer underscreening in difficult-to-reach individuals, and potential opportunities for interventions targeting multiple care gaps (eg, with guideline-approved CRC self-screening options,^52^ interventions promoting home-based screening for both cervical and CRC cancer could be beneficial). We previously evaluated whether the HOME intervention impacted subsequent receipt of other recommended screening. Although the intervention had no impact on mammography or CRC screening compared with usual care, intervention group participants who completed a kit were more likely to receive mammography or CRC screening than those who remained unscreened for cervical cancer.^53^

We also saw a difference in intervention effect by age, with the largest relative and absolute increases in screening in the oldest groups, although the differences were not statistically significant. Additionally, compared with younger participants, older participants who were mailed kits were also significantly more likely to screen by kit vs in-clinic. US population-level^15^ and KPWA^14^ data show that among individuals aged 30 to 65 years, cervical cancer screening adherence declines with increasing age. HPV self-sampling may address barriers in older individuals related to negative prior screening experiences,^54,55^ embarrassment,^55,56^ or discomfort from speculum examinations that may be exacerbated by age-related vaginal atrophy and menopause.^48,57^ Associations between age and response to mailed HPV kits have been inconsistent across international studies, highlighting the importance of setting-specific data to inform implementation strategies.^34,35,49,50,58,59,60,61^

In the US, there are documented disparities in cervical cancer screening rates by race and ethnicity^1,22,62,63,64,65^ that likely contribute to disparities in cervical cancer incidence and mortality.^66^ Screening barriers and patient-reported reasons for underscreening also vary by race and ethnicity, which may contribute to differences in intervention effectiveness across subpopulations.^1,65,67^ In HOME, neither race nor ethnicity modified the intervention effect on screening uptake. Within the intervention group, however, differences were seen. Kit uptake was highest in White participants and lowest in Native Hawaiian/other Pacific Islander and American Indian/Alaska Native participants. Additionally, the proportion of screened participants who returned kits ranged from a low of 24.2% in Native Hawaiian/other Pacific Islander participants to a high of 47.6% in White participants. Results suggest opportunities to increase self-sampling uptake in racial and ethnic subgroups by tailoring outreach strategies and/or kit components. Other studies reported differences in acceptance or uptake of self-sampling by race or ethnicity.^48,57,68^ There is also heterogeneity within racial and ethnic groups that we were unable to evaluate, but that may contribute to differences in screening rates and intervention effectiveness. Additionally, lower cervical cancer screening rates have been associated with limited English proficiency (in majority English-speaking countries) and recent immigrant status.^65,69,70^ HOME excluded individuals who require a language interpreter (kit materials were in English only), precluding comparisons between those with and without limited English proficiency. In addition, we did not have data on country of birth (and the language restriction likely would have excluded most recent immigrants).

Intervention effectiveness did not differ by BMI, tobacco use, or comorbidities, and among those mailed kits, no significant differences in kit vs in-clinic screening uptake were observed by these patient characteristics. Higher BMI,^14,17^ tobacco use,^14,18^ and comorbidities^14,25,26^ are associated with underscreening, yet our results suggest HPV self-sampling can effectively increase screening participation in patients with these characteristics. Additionally, results did not vary by clinic travel time (with the caveat that most participants lived in urban settings).

### Strengths and Limitations

A strength of this study is the pragmatic trial design,^32,71^ including integration of the intervention into existing clinical protocols. Furthermore, to reduce participation bias and enhance generalizability, we enrolled all individuals identified as eligible through EMR data under a waiver of consent. Only 1.2% of intervention group participants opted out of medical record review and were excluded from this analysis of patient characteristic modifiers.

Mailing HPV kits through a research study rather than as standard care may have positively or negatively impacted screening uptake, and impacts may have been differential by patient characteristics. Additionally, the recommendation to attend in-clinic screening even after a negative self-sampling result may have negatively impacted screening uptake, with potential for differential impacts by patient characteristics. In addition to English language proficiency and country of birth, we were also unable to evaluate household income and education level due to limitations of EMR data. We used geocoded US Census tract household income, which has limitations as a proxy for individual-level household income.^72^ Additionally, there is potential for misclassification of patient characteristics derived from EMR data, especially race and ethnicity.^73^

## Conclusions

Within a US health care system, a mailed HPV kit intervention was associated with an 8.9% increase in screening compared with usual care, with clinically important increases observed across all sociodemographic and health characteristics evaluated. Although we identified some patient characteristics that modified intervention effectiveness, there were notable differences when comparing relative vs absolute effects. Absolute effects may be particularly relevant for health care system decision making around how to allocate resources for maximal impact on screening rates and cervical cancer prevention in high-risk groups. We also noticed differences in kit use by several patient characteristics, highlighting opportunities to optimize HPV self-sampling for priority subgroups. Most individuals in the intervention group remained underscreened, and uptake was particularly low (≤10%) in patient subgroups defined by long duration of cervical cancer underscreening, Native Hawaiian/other Pacific Islander or American Indian/Alaska Native race, and nonadherence to breast or CRC screening. Tailored interventions to reduce cervical cancer underscreening in these priority populations are needed.

## Notes

Supplement 1. **eAppendix.** Trial Protocol

Supplement 2.
Data Sharing Statement
