# Supplementary material for: Effect of Patient Characteristics on Uptake of Screening Using a Mailed Human Papillomavirus Self-sampling Kit: A Secondary Analysis of a Randomized Clinical Trial
Source: JAMA Netw Open. 2022 Nov 30;5(11):e2244343. doi: 10.1001/jamanetworkopen.2022.44343 (PMC9713609; doi:10.1001/jamanetworkopen.2022.44343)
Supplement: Supplement 1. — eAppendix. Trial Protocol [file jamanetwopen-e2244343-s001.pdf]

Supplemental Online Content

Winer RL, Lin J, Tiro JA, et al. Effect of patient characteristics on uptake of screening using a mailed human papillomavirus self-sampling kit: a secondary analysis of a randomized clinical trial. *JAMA Netw Open*. 2022;5(11):e2244343. doi:10.1001/jamanetworkopen.2022.44343

eAppendix. Trial Protocol

This supplemental material has been provided by the authors to give readers additional information about their work.

© 2022 Winer RL et al. *JAMA Network Open.*

ClinicalTrials.gov Identifier: NCT02005510

OFFICIAL TITLE: Randomized Trial of In-Home Cervical Cancer Screening in Underscreened Women

SHORT TITLE: HOME Study

Coordinating Center: University of Washington

Principal Investigator: *Rachel L. Winer*
Department of Epidemiology
School of Public Health
University of Washington
Seattle, Washington, U.S.A.

Co-Investigator: *Diana S.M. Buist*
Kaiser Permanente Washington Health Research Institute
Seattle, Washington, U.S.A.

Study Coordinator: *Tara Beatty*
Kaiser Permanente Washington Health Research Institute
Seattle, Washington, U.S.A.

Study Exempt from IDE requirements

On September 12, 2013 (Q130922 Study Determination request) FDA determined intervention is a nonsignificant risk (NSR) device as does not meet definition of a significant risk (SR) device under §812.3(m) of the investigational device exemptions (IDE) regulation (21 CFR 812).

Final Protocol Date: *March 17, 2015*

Protocol Revision Dates: *March 17, 2015*

Initial Protocol Date: *January 7, 2014*

© 2022 Winer RL et al. *JAMA Network Open.*

SCHEMA, FIGURE 1

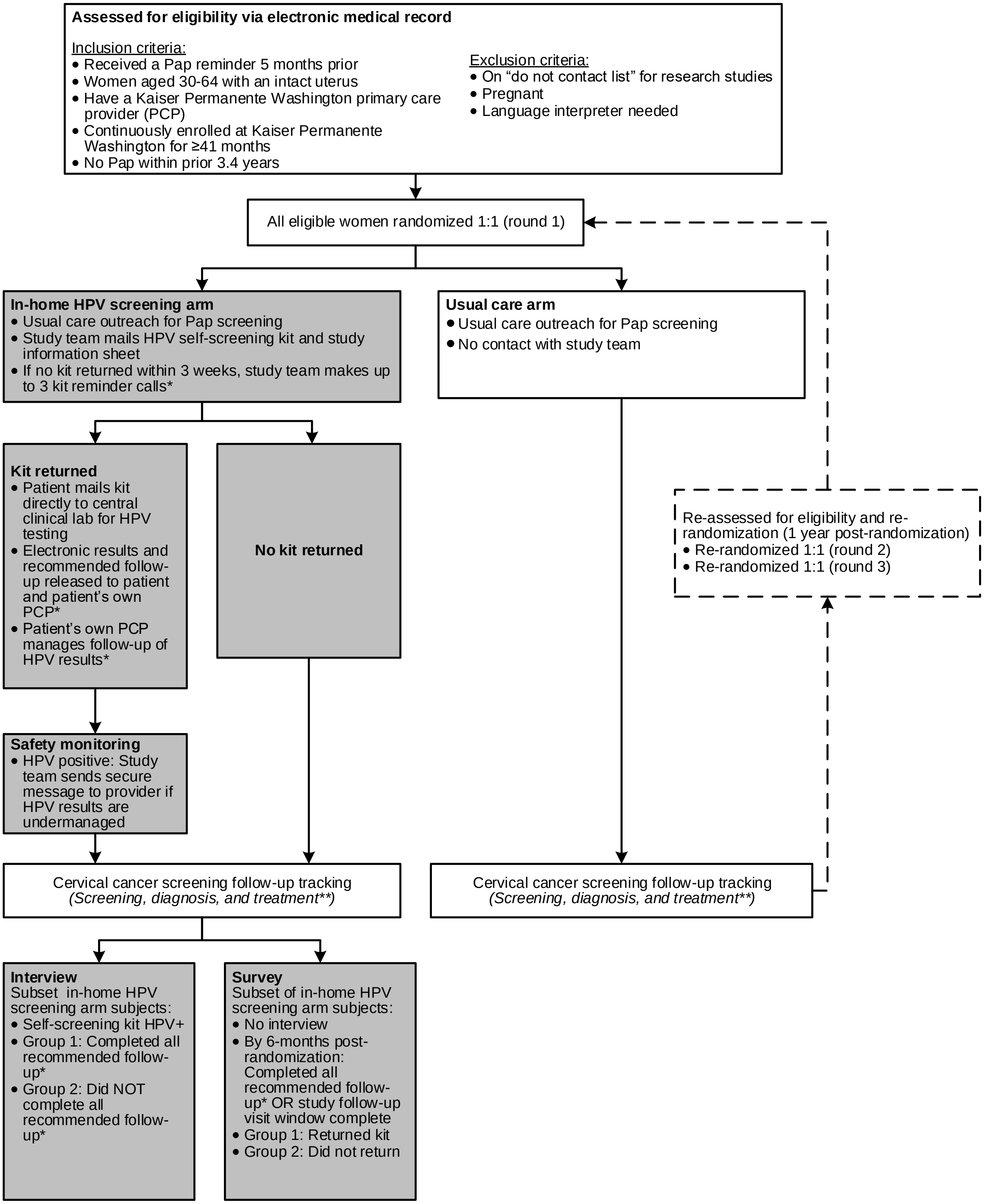

* Mirrors clinical system outreach or follow-up procedures
** See Figure 2 for diagram of cervical cancer screening, diagnosis, and treatment outcome definitions and time windows

© 2022 Winer RL et al. *JAMA Network Open.*

SCHEMA, FIGURE 2

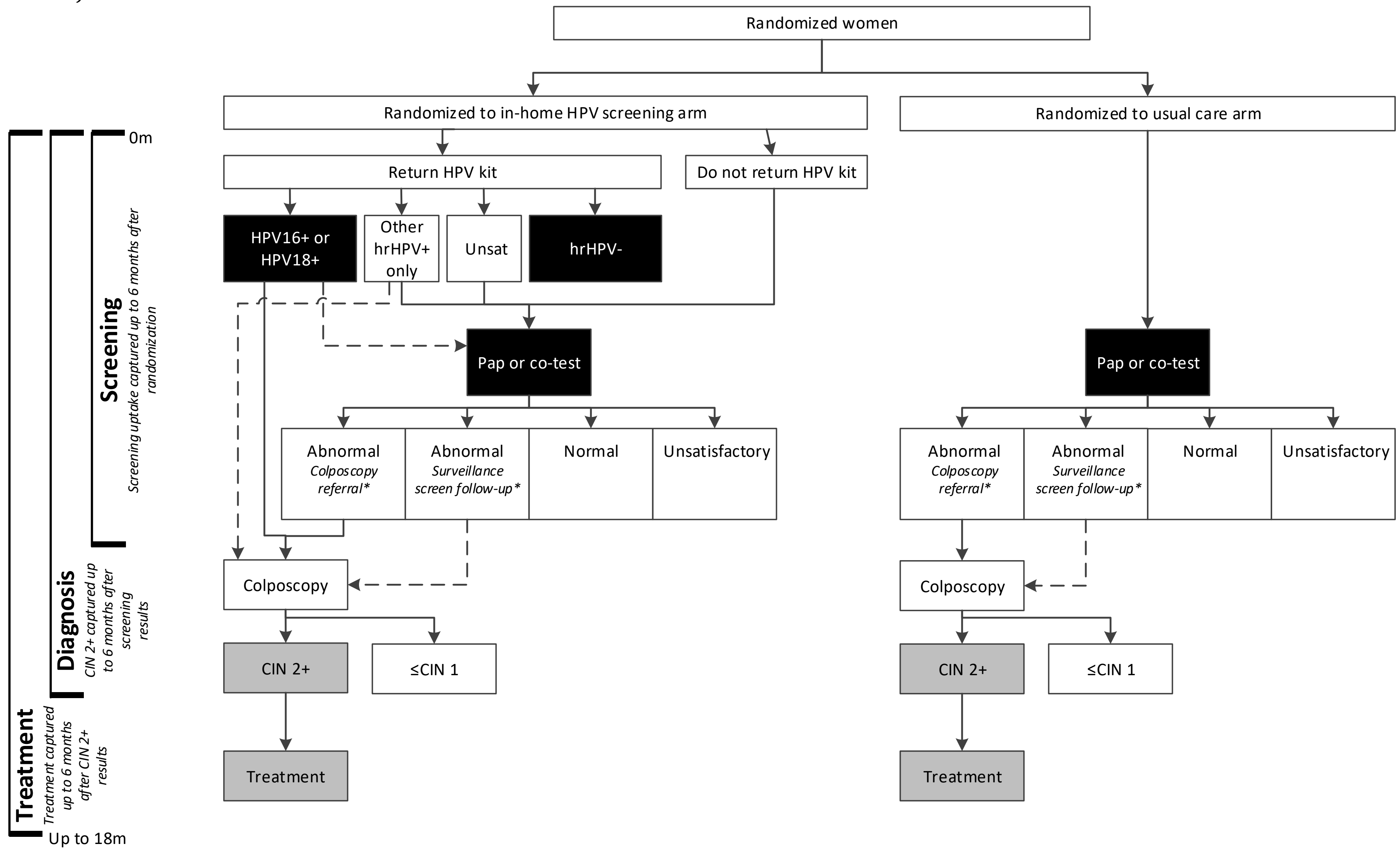

Black boxes represent screening uptake outcomes

Grey boxes represent diagnosis and treatment outcomes

Dashed lines represent non guideline-recommended management

* Asterisk notes follow-up per current national guidelines (2012 cervical cancer screening [1] and 2013 abnormal result management guidelines [2]), i.e.,
- Colposcopy referral: Pap and/or HPV result of ≥LSIL or ASC-US & HPV+, or HPV 16/18+
- Surveillance screen follow-up: Pap and/or HPV result of ASC-US or LSIL & HPV–, or Pap– & HPV+

Abbreviations: CIN, cervical intraepithelial neoplasia; LSIL, low-grade squamous intraepithelial lesion; ASC-US, atypical squamous cells of undetermined significance; HPV, human papillomavirus; hrHPV, high risk human papillomavirus

© 2022 Winer RL et al. *JAMA Network Open.*

SUMMARY OF CHANGES – PROTOCOL

#	Date	Change
1.	March 17, 2015	One-year post-randomization, control group participants re-assessed for eligibility and re-randomized.
2.		
3.		
4.		
5.		

© 2022 Winer RL et al. *JAMA Network Open.*

TABLE OF CONTENTS

SCHEMA, Figure 1 3

SCHEMA, Figure 2 4

SUMMARY OF CHANGES – Protocol 5

1. OBJECTIVES 7
   1.1 Primary Objectives 7
   1.2 Secondary Objectives 7

2. BACKGROUND 7
   2.1 Study Disease(s) 7
   2.2 Rationale 8

3. PATIENT SELECTION 8
   3.1 Eligibility Criteria 8
   3.2 Exclusion Criteria 9
   3.3 Inclusion of Women and Minorities 9
   3.4 Inclusion of Children 9

4. STUDY PROCEDURES 9
   4.1 Subject Recruitment and Screening 9
   4.2 Procedures 10
   4.3 Early Termination 11

5. STATISTICAL CONSIDERATIONS 11
   5.1 Sample Size 12
   5.2 Analysis Plans 12

6. ADVERSE EVENTS: REPORTING REQUIREMENTS 13
   6.1 Determination of Study Risk 13
   6.2 Reporting Adverse Events 14
   6.3 Reporting the Intensity of an Adverse Event 14
   6.4 Reporting the Relationship of an Adverse Event to intervention 14

7. STUDY OVERSIGHT AND DATA REPORTING / REGULATORY REQUIREMENTS 15
   7.1 Protocol Review 15
   7.2 Informed Consent 15
   7.3 Changes to Protocol 15
   7.4 Data and Safety Monitoring Plan 15

8. REFERENCES 17

APPENDIX A NCI Common Terminology Criteria for Adverse Events CTC AE v3.0 19

© 2022 Winer RL et al. *JAMA Network Open.*

OBJECTIVES

1.1 Primary Objectives

1.1.1 Histologically-diagnosed CIN 2+ within 6 months after an abnormal screening result (up to 12 months post-randomization)

1.1.2 Treated CIN 2+ within 6 months after diagnosed CIN 2+ (up to 18 months post-randomization)

1.2 Secondary Objectives

1.2.1 Screening uptake within 6 months after randomization

1.2.2 Abnormal screening result within 6 months after randomization

1.2.3 Predictors of screening uptake and intervention effectiveness through administrative data

1.2.4 Experiences and attitudes associated with in-home HPV testing uptake (through surveys)

1.2.5 Experiences and attitudes associated with follow-up of positive in-home HPV testing results (through semi-structured interviews)

BACKGROUND

2.1 Study Disease(s)

Despite large-scale efforts to encourage routine Papanicolaou (Pap) screening for cervical cancer prevention, 20%-30% of U.S. women ≥30 years of age have not been screened in the past 3 years.[3,4] More than half of all cervical cancers in the U.S. are diagnosed in these unscreened or underscreened women.[5–7] 2012 national guidelines identify increasing screening coverage as the #1 research priority for reducing cervical cancer-related morbidity and mortality.[1] Innovative strategies that eliminate the need for clinic-based primary screening could effectively improve screening compliance while maintaining high-quality care.

Studies have demonstrated improved sensitivity and cost effectiveness for detecting pre-cancerous cervical neoplasia grade 2 to 3, carcinoma in situ, and invasive cervical cancer (hereafter referred to as CIN 2,3+) from Pap co-testing with FDA-approved tests for high-risk (hr) HPV infection compared to Pap screening alone for women ≥30 years of age.[8,9] While this approach is endorsed by all major U.S. guidelines as a preferred or acceptable screening strategy[1,10] and has advantages over traditional Pap screening, co-testing is no more likely to attract women who delay attending clinic-based screening. There is growing interest in an alternative primary screening strategy – hrHPV screening followed by triage of women with hrHPV+ test results to cytology, with studies suggesting that such a strategy could be cost-

© 2022 Winer RL et al. *JAMA Network Open.*

effective and sensitive for detecting CIN 2,3+.[11,12] If samples for hrHPV screening could be self-collected at home (with in-clinic follow-up of hrHPV-positive women), the need for in-clinic screening could be eliminated for a majority of women.

2.2 Rationale

Despite the availability of highly effective prophylactic hrHPV vaccines, screening remains a necessary priority for cervical cancer prevention.[1,10] Current vaccines do not protect against all hrHPV types, nor do they protect women already infected with vaccine-types from developing cervical neoplasia.[13] Furthermore, because these vaccines have only been available since 2006, are only recommended for women ≤26 years of age,[14] and have not been widely used even among the recommended target population,[15] the majority of women ≥30 years of age (the age group at highest risk for cervical cancer) are unvaccinated. Although Pap screening programs have been highly effective in reducing cervical cancer rates over the past 50 years, a significant portion of U.S. women do not participate in regular Pap screening; 20%-30% of U.S. women ≥30 years of age have not been screened in the past three years.[3,4] These are the women at highest risk for cervical cancer, as over half of the 12,000 cervical cancers diagnosed in the U.S. each year[16] are in women who have not been screened in the past three years.[5–7] Reaching underscreened women is a top national priority for reducing disparities in cervical cancer prevention; in fact, the 2012 joint cervical cancer screening guidelines state that the #1 research priority is to increase screening coverage.[1] There is a significant need for targeted, innovative interventions that increase screening participation and adherence to recommended screening intervals, while maintaining high quality care. The joint guidelines advocate for novel programs incorporating self-sampling for hrHPV testing, and evaluation of the "scale-up, implementation, and acceptability of such programs[17]" targeting underscreened women.[1] Our proposed RCT is directly responsive to this national recommendation, and will provide definitive evidence-based data on the ability of an in-home programmatic HPV screening outreach strategy to enhance early detection of cervical neoplasia and improve screening compliance. It is likely that this innovative study could change the ways in which women participate in cervical cancer screening programs. Furthermore, we will investigate patient experiences and attitudes that are associated with in-home HPV screening uptake and complete follow up of hrHPV+ test results. The latter is particularly important for understanding adherence to the continuum of cervical cancer prevention, from screening through treatment. While the majority of cervical cancers are attributable to lack of screening, up to 13% of cervical cancers diagnosed in fully-insured women are attributable to delayed follow up of abnormal Pap results.[7,18,19]

PATIENT SELECTION

3.1 Eligibility Criteria

3.1.1 Female sex

3.1.2 30 years to 64 years of age

© 2022 Winer RL et al. *JAMA Network Open.*

3.1.3 Have a primary care provider at Kaiser Permanente Washington

3.1.4 Received annual "birthday letter" with Pap screening reminder 5 months earlier

3.1.5 No Pap test in the past 3.4 years

3.1.6 Continuously enrolled at Kaiser Permanente Washington for at least 3.4 years

3.1.7 No hysterectomy

3.2 Exclusion Criteria

3.2.1 Currently pregnant

3.2.2 Language interpreter needed

3.2.3 On "do not contact list" for research studies

3.3 Inclusion of Women and Minorities

The scientific objective of the proposed research is to study the ability of an in-home programmatic HPV screening outreach strategy to enhance early detection of cervical neoplasia and improve screening compliance. Because cervical cancer only affects women, our entire study population will be composed of women.

Race and ethnicity are not eligibility requirements for participation in our study. We will include all minorities that are Kaiser Permanente Washington members and meet our study eligibility requirements. The ethnic/racial composition of our study population will therefore largely reflect the ethnic/racial composition of Kaiser Permanente Washington women members. The projected proportions of participants from different ethnic/racial backgrounds are based on the composition of Kaiser Permanente Washington members (See Planned Enrollment Report table in Section 5.1).

3.4 Inclusion of Children

We are not enrolling women younger than age 21 in the current study. All current guideline recommendations (2012) are that cervical cancer screening should begin at age 21 years (regardless of sexual history). Screening before age 21 should be avoided because women less than 21 years old are at very low risk of cancer. Screening these women may lead to unnecessary and harmful evaluation and treatment.

STUDY PROCEDURES

4.1 Subject Recruitment and Screening

© 2022 Winer RL et al. *JAMA Network Open.*

4.1.1 Primary objectives, and secondary objectives one (1.2.1), two (1.2.2), and three (1.2.3)

Eligible women will be identified using electronic medical record (EMR) data; all eligible women will be enrolled under a waiver of consent. The study programmer will use SAS software built in simple random sample procedure to randomly allocate participants 1:1 to the intervention arm or the control arm over a 2.5 year recruitment period. One-year post-randomization, control arm participants will be re-assessed for eligibility and re-randomized.

Women in the control arm will receive usual care reminders to attend Pap screening. Women randomized to the usual care arm will not receive any study-related interventions or contact from the study team.

Women in the intervention group will receive usual care plus a mailed HPV self-sampling kit with a pre-paid envelope addressed to Kaiser Permanente Washington to return the kit to the central clinical laboratory. The mailing will include an invitation letter, research information sheet, and materials for self-collecting and returning a sample. Because home HPV self-screening is not standard of care in the US, the invitation letter will advise women to receive routine Pap tests, regardless of whether they select to complete HPV self-sampling. Women will be informed participation is voluntary and provided with a telephone number to call with questions or to "opt-out" of having their individual-level medical record data used for research. To mirror Kaiser Permanente Washington prevention outreach protocols, if the kit is not returned within three weeks, study staff will make up to three reminder calls.

4.1.2 Secondary objective four (1.2.4), survey

We will mail survey invitation letters to intervention arm participants six months after trial randomization. We will sample two groups based on kit return status, using EMR data to identify and recruit "kit returners" and "non-returners". Invitation letters will ask women to complete a 5-10 minute web survey about their experience with a "health screening kit" mailed 6 months prior.

4.1.3 Secondary objective five (1.2.5), interview

Potential interview participants will be identified 1 to 2 weeks after completing all recommended diagnostic follow-up or treatment, or after they are no longer being assessed for main trial outcomes if recommended follow-up was not completed. An invitation letter and information sheet will be mailed with a telephone number to opt-out. If the potential participant does not opt-out, an interviewer will call a few days later to conduct a 15–20 minute interview.

4.2 Procedures

4.2.1 Medical records history

We will obtain the following from subject medical records: (1) cervical cancer screening, diagnosis, and treatment, (2) medical information related to cervical cancer risk factors, and (3) demographic information. For example, doctor visits and lab tests, age, ethnic background and

© 2022 Winer RL et al. *JAMA Network Open.*

other demographics, tobacco use, diagnoses, and disease history.

4.2.2 Clinical outcomes

We will obtain cervical cancer screening, diagnosis, and treatment results from subject medical records up to 18 months post-randomization.

4.2.3 Self-collect vaginal specimen for HPV testing

Subjects randomized to the intervention arm (in-home screening) will be asked to wash their hands and then push a polyester swab into their vagina as far as they can with no pain. Rotate three times and take the swab out. Put the swab in a specimen cup. Repeat the sample collection on a second swab. Place the specimen in a return mailer and mail the sample to the Kaiser Permanente Washington central laboratory.

4.2.4 Survey

Subjects will be asked questions about their attitudes and knowledge of the self-sampling kit previously mailed to them, and preference for at-home screening compared vs. clinic-based screening, and their knowledge of cervical cancer and cervical cancer screening.

4.2.5 Interview

Subjects will be asked about their experience and thoughts about the self-sampling kit previously mailed to them, Pap testing, their test preferences, their knowledge of cervical cancer and cervical cancer screening, and their contacts with their doctor or health care team.

4.3 Early Termination

Any subjects experiencing a serious adverse event felt to be related to study procedures should be withdrawn from the study. Any subject withdrawing their consent to participate in the study or their authorization to use their protected health information will be withdrawn from the study.

All subjects randomized into the study will be included in the final study analyses. Subjects may be withdrawn from the study if:

1) Voluntary patient withdrawal;

Reasons why subjects are discontinued from the clinical trial will be documented on the study termination tracking log.

STATISTICAL CONSIDERATIONS

© 2022 Winer RL et al. *JAMA Network Open.*

5.1 Sample Size

PLANNED ENROLLMENT REPORT

ANTICIPATED/PLANNED ENROLLMENT for ENTIRE STUDY: Number of Participants (*must provide exact numbers. i.e. no range)*			
**Ethnic Categories**	**Sex/Gender**		
	Females	Males	Total
Hispanic or Latino	880	0	880
Not Hispanic or Latino	16,711	0	16,711
**Ethnic Categories: Total of All Participants**	17,591	0	17,591
**Racial Categories**			
American Indian/Alaska Native	705	0	705
Asian	1,406	0	1,406
Native Hawaiian or Other Pacific Islander	176	0	176
Black or African American	880	0	880
White	14,424	0	14,424
**Racial Categories: Total of All Participants**	17,591	0	17,591

Estimated based on the number and racial and ethnic distribution of underscreened women in 2011 in Kaiser Permanente Washington

5.2 Analysis Plans

5.2.1 Analysis plan relevant to primary objectives and secondary objectives one (1.2.1), two (1.2.2), and three (1.2.3)

Data will be analyzed based on the intention-to-treat principle. Denominators for each arm will generally include all women randomized to that arm, minus the women identified post-randomization as ineligible. For diagnosed and treated CIN 2+, abnormal screening results, and screening uptake, we will compare the proportion of outcomes detected in the intervention arm to the proportion detected in the usual care arm and estimate relative risks using log-binomial regression. Robust variances estimates will be used to account for within-subject correlation due to re-randomized subjects contributing more than one observation period. If differences are observed in the distribution of EMR-derived subject characteristics across arms despite randomization, we will adjust for the relevant covariates in the regression models. Subject characteristics of interest include age, race, ethnicity, length of health plan enrollment before randomization, etc.

To evaluate predictors of screening uptake, we will use log-binomial regression to estimate the effects of subject characteristics on the probability of screening uptake. To test for effect modification by randomization arm (i.e., to test if home HPV screening is more effective at increasing uptake than usual care for subgroups of women), we will test characteristic-by-randomization arm interaction terms using log-binomial regression comparing the relative risk of screening uptake in the intervention arm relative to the usual care arm by characteristics of

© 2022 Winer RL et al. *JAMA Network Open.*

interest.

In an exploratory analysis, positive predictive value (PPV) of an abnormal screening test for detecting CIN 2+ will be estimated within each arm. The denominator will include women who receive an abnormal screening result within 6 months of randomization that warrants referral to colposcopy, and the numerator will include women with diagnosed CIN 2+. We will also calculate PPV restricting the denominator to women who receive colposcopy within 6 months of the abnormal screening result.

5.2.2 Analysis plans relevant to secondary objectives four (1.2.4) and five (1.2.5)

Through surveys and in-depth interviews of women in the in-home HPV screening arm, we will identify patient experiences and attitudes that are associated with in-home HPV testing uptake and complete follow up of hrHPV+ test results. We will describe frequency distributions of responses to survey questions about patient experiences and attitudes (aligned with our conceptual model–adapted from Andersen-Aday[20–22] & Proctor[23]). We will transcribe, code and analyze the semi-structured interviews to examine experiences and attitudes related to timely completion of follow up of hrHPV+ results. Together with the system-level impact data, these results will provide important information about how multi-level systems can support timely follow up for hrHPV+ women, and areas for additional educational intervention.

ADVERSE EVENTS: REPORTING REQUIREMENTS

6.1 Determination of Study Risk

This is a minimal risk study where the medical intervention has similar potential adverse events as women undergoing standard clinical procedures such as Pap, human papillomavirus (HPV) or other sexually transmitted disease testing. Based on our own previous and ongoing studies and numerous other prior studies involving self-collected vaginal swabs, we expect adverse events will be rare and minor in severity. The study questionnaires and interviews also present minimal risk to subjects as they do not address highly sensitive information. As such, the principal investigator will continuously monitor adverse events as they are reported to the study hotline and also through women's primary care teams. Both reviewing institutional review boards for this study (University of Washington and Kaiser Permanente Washington) have ruled in agreement with this assessment.

Discomfort and light bleeding are the expected adverse events (AEs). In our previous University of Washington studies of in-home HPV testing, light bleeding was reported by a small minority of subjects who used in-home HPV test kits (less frequent than bleeding from standard Pap testing). We have no plan for stopping rules due to bleeding because we are only capturing AEs through self-report to the study hotline or primary care teams, and do not expect that the number of reports of bleeding would exceed the frequency of bleeding from standard Pap testing. All adverse events will be continuously monitored by the Principal Investigator.

Toxicities and adverse events will be assessed using the NCI Common Toxicity Criteria for Adverse Events v3.0 (CTCAE; see Appendix A).

© 2022 Winer RL et al. *JAMA Network Open.*

6.2 Reporting Adverse Events

All adverse events, whether solicited or spontaneous, must be documented in the adverse event (AE) case report form (CRF).

Adverse events will be reported by patients via the study telephone hotline, or via report to their Kaiser Permanente provider (providers will report AEs to study staff). All AEs reported to study staff, regardless of causality, must be recorded immediately in the AE CRF.

The Principal Investigator will designate a medical monitor that will be responsible for following AEs that are serious or that cause the patient to discontinue before completing the study, through an appropriate health care option. The patient should be followed until the event resolves or stabilizes. Frequency of follow-up is at the discretion of the medical monitor. The medical monitor must follow the clinical course of each AE until resolution or stabilization. Serious AEs ongoing at the end of the study period must be followed up to final outcome.

6.3 Reporting the Intensity of an Adverse Event

The intensity of an AE will be described and graded per NCI Common Terminology Criteria for Adverse Events CTC AE v3.0 (http://ctep.cancer.gov/protocolDevelopment/electronic_applications/docs/ctcaev3.pdf) .

6.4 Reporting the Relationship of an Adverse Event to intervention

The medical monitor will determine the assessment of the causal relationship of the event to study intervention using the following guidelines:

- **Attribution** of the AE:
  - Definite – The AE *is clearly related* to the study treatment.
  - Probable – The AE *is likely related* to the study treatment.
  - Possible – The AE *may be related* to the study treatment.
  - Unlikely – The AE *is doubtfully related* to the study treatment.
  - Unrelated – The AE *is clearly NOT related* to the study treatment.

All adverse events, regardless of severity, will be classified as expected or unexpected and reported to the Kaiser Permanente Washington Human Subjects Review Committee, per current Kaiser Permanente Washington Human Subjects Review Committee Incident Guidelines.

© 2022 Winer RL et al. *JAMA Network Open.*

STUDY OVERSIGHT AND DATA REPORTING / REGULATORY REQUIREMENTS

7.1 Protocol Review

The protocol and informed consent forms for this study must be reviewed and approved in writing by a properly constituted independent Ethics Committee (EC) or Institutional Review Board (IRB) prior to any patient being registered on this study.

7.2 Informed Consent

7.2.1 Primary objective and secondary objectives one (1.2.1), two (1.2.2), and three (1.2.3)

All consent conduct in compliance with Code of Federal Regulations, Title 45, Part 46 (45 CFR part 46). To reduce participation bias, all eligible women will be enrolled into the trial under a waiver of consent. Informed consent of intervention arm kit recipients will be per a waiver of documentation of consent. Intervention arm women will have the ability to opt-out of having their individual-level medical record data used in the research, but passive consent will be utilized which will significantly enhance the generalizability of the findings.

7.2.2 Secondary objectives four (1.2.4) and five (1.2.5) (surveys and in-depth interviews of women in the in-home HPV screening arm)

In compliance with 45 CFR part 46, informed consent will be obtained from all women via a waiver of documentation of consent.

7.3 Changes to Protocol

Any modification of this protocol must be approved by the Principal Investigator and approved by the IRB(s), before the revision or amendment may be implemented. The only circumstance in which the amendment may be initiated without regulatory approval is for a change necessary to eliminate an apparent and immediate hazard to the patient. In that event, the investigator must notify the IRB in writing per current IRB rules.

7.4 Data and Safety Monitoring Plan

An interim data look at the kit return rate in the intervention arm will be reviewed by limited study staff 6 weeks after reaching 50% of the expected target accrual; a kit return rate of >10% is set as the threshold for continuing the trial because a lower return rate will make the intervention not clinically viable.

Study staff involved in interim data activities will not be involved in any scientific decisions about modifications to the study protocol, but may consult with an external scientific advisory committee if review of study data raises any potential ethical concerns. Membership on the

© 2022 Winer RL et al. *JAMA Network Open.*

external scientific committee will include a Kaiser Permanente Washington Health Research Institute biostatistician and a University of Washington clinician. The scientific leadership committee, comprised of the Principal Investigator, project PhD biostatistician, and one additional co-investigator, will be blinded to all primary and secondary outcomes analyses until 6 months after the last subject is enrolled.

© 2022 Winer RL et al. *JAMA Network Open.*

REFERENCES

1. Saslow D, Solomon D, Lawson HW, et al. American Cancer Society, American Society for Colposcopy and Cervical Pathology, and American Society for Clinical Pathology screening guidelines for the prevention and early detection of cervical cancer. *CA Cancer J Clin*. 2012;62(3):147-172. doi:10.3322/caac.21139

2. Massad LS, Einstein MH, Huh WK, et al. 2012 updated consensus guidelines for the management of abnormal cervical cancer screening tests and cancer precursors. *Obstet Gynecol*. 2013;121(4):829-846. doi:10.1097/AOG.0b013e3182883a34

3. *Health US, 2009: With Special Feature on Medical Technology*. Hyattsville, MD: National Center for Health Statistics; 2010.

4. *The State of Healthcare Quality 2010*. The National Committee for Quality Assurance; 2010.

5. Kinney W, Sung HY, Kearney KA, Miller M, Sawaya G, Hiatt RA. Missed opportunities for cervical cancer screening of HMO members developing invasive cervical cancer (ICC). *Gynecol Oncol*. 1998;71(3):428-430. doi:10.1006/gyno.1998.5135

6. Janerich DT, Hadjimichael O, Schwartz PE, et al. The screening histories of women with invasive cervical cancer, Connecticut. *Am J Public Health*. 1995;85(6):791-794. doi:10.2105/ajph.85.6.791

7. Leyden WA, Manos MM, Geiger AM, et al. Cervical cancer in women with comprehensive health care access: attributable factors in the screening process. *J Natl Cancer Inst*. 2005;97(9):675-683. doi:10.1093/jnci/dji115

8. Goldie SJ, Kim JJ, Wright TC. Cost-effectiveness of human papillomavirus DNA testing for cervical cancer screening in women aged 30 years or more. *Obstet Gynecol*. 2004;103(4):619-631. doi:10.1097/01.AOG.0000120143.50098.c7

9. Wright TC, Schiffman M, Solomon D, et al. Interim guidance for the use of human papillomavirus DNA testing as an adjunct to cervical cytology for screening. *Obstet Gynecol*. 2004;103(2):304-309. doi:10.1097/01.AOG.0000109426.82624.f8

10. Moyer VA, U. S. Preventive Services Task Force. Screening for cervical cancer: U.S. Preventive Services Task Force recommendation statement. *Ann Intern Med*. 2012;156(12):880-891, W312. doi:10.7326/0003-4819-156-12-201206190-00424

11. Balasubramanian A, Kulasingam SL, Baer A, et al. Accuracy and cost-effectiveness of cervical cancer screening by high-risk human papillomavirus DNA testing of self-collected vaginal samples. *J Low Genit Tract Dis*. 2010;14(3):185-195. doi:10.1097/LGT.0b013e3181cd6d36

12. Cuzick J, Arbyn M, Sankaranarayanan R, et al. Overview of human papillomavirus-based and other novel options for cervical cancer screening in developed and developing

© 2022 Winer RL et al. *JAMA Network Open.*

countries. *Vaccine*. 2008;26 Suppl 10:K29-41. doi:10.1016/j.vaccine.2008.06.019

13. Markowitz LE. HPV vaccines prophylactic, not therapeutic. *JAMA*. 2007;298(7):805-806. doi:10.1001/jama.298.7.805

14. Markowitz LE, Dunne EF, Saraiya M, et al. Quadrivalent Human Papillomavirus Vaccine: Recommendations of the Advisory Committee on Immunization Practices (ACIP). *MMWR Recomm Rep Morb Mortal Wkly Rep Recomm Rep*. 2007;56(RR-2):1-24.

15. Advisory Committee on Immunization Practices. Recommended adult immunization schedule: United States, 2012. *Ann Intern Med*. 2012;156(3):211-217. doi:10.7326/0003-4819-156-3-201202070-00388

16. U.S. Cancer Statistics Working Group. *United States Cancer Statistics: 1999–2007 Incidence and Mortality Web-Based Report*. Atlanta, GA: Department of Health and Human Services http://www.cdc.gov/uscs.

17. World Health Organization, Department of Reproductive Health and Research. *Practical Guidance for Scaling Up Health Service Innovations*. Geneva, Switzerland: World Health Organization; 2009.

18. Stuart GC, McGregor SE, Duggan MA, Nation JG. Review of the screening history of Alberta women with invasive cervical cancer. *CMAJ Can Med Assoc J J Assoc Medicale Can*. 1997;157(5):513-519.

19. Sung HY, Kearney KA, Miller M, Kinney W, Sawaya GF, Hiatt RA. Papanicolaou smear history and diagnosis of invasive cervical carcinoma among members of a large prepaid health plan. *Cancer*. 2000;88(10):2283-2289.

20. Aday LA, Andersen R. A framework for the study of access to medical care. *Health Serv Res*. 1974;9(3):208-220.

21. Andersen RM. Revisiting the behavioral model and access to medical care: does it matter? *J Health Soc Behav*. 1995;36(1):1-10.

22. Yabroff KR. Interventions to improve cancer screening: commentary from a health services research perspective. *Am J Prev Med*. 2008;35(1 Suppl):S6-9. doi:10.1016/j.amepre.2008.04.006

23. Proctor E, Silmere H, Raghavan R, et al. Outcomes for implementation research: conceptual distinctions, measurement challenges, and research agenda. *Adm Policy Ment Health*. 2011;38(2):65-76. doi:10.1007/s10488-010-0319-7

© 2022 Winer RL et al. *JAMA Network Open.*

APPENDIX A NCI COMMON TERMINOLOGY CRITERIA FOR ADVERSE EVENTS CTC AE V3.0

Adverse events will be assessed using the NCI Common Toxicity Criteria for Adverse Events v3.0 (CTCAE). A copy can be downloaded from the CTEP home page.

http://ctep.cancer.gov/protocolDevelopment/electronic_applications/ctc.htm

© 2022 Winer RL et al. *JAMA Network Open.*
