# Supplementary material for: Effect of Patient Characteristics on Uptake of Screening Using a Mailed Human Papillomavirus Self-sampling Kit: A Secondary Analysis of a Randomized Clinical Trial
Source: JAMA Netw Open. 2022 Nov 30;5(11):e2244343. doi: 10.1001/jamanetworkopen.2022.44343 (PMC9713609; doi:10.1001/jamanetworkopen.2022.44343)
Supplement: Supplement 2. — Data Sharing Statement [file jamanetwopen-e2244343-s002.pdf]

Data Sharing Statement

Winer. Effect of Patient Characteristics on Uptake of Screening Using a Mailed Human Papillomavirus Self-sampling Kit. *JAMA Netw Open*. Published November 30, 2022. doi:10.1001/jamanetworkopen.2022.44343

Data
Data available: Yes
Data types: Deidentified participant data, Data dictionary
How to access data: Requests for data must be sent to rlw@uw.edu.
When available: With publication

Supporting Documents
Document types: None

Additional Information
Who can access the data: Data will be made available to researchers whose proposed use of the data has been approved.
Types of analyses: Data will be made available for a specified purpose.
Mechanisms of data availability: Data will be made available without investigator support to researchers with adequate resources to cover the regulatory and data sharing costs. Data will be made available after approval of a concept proposal aligned with current data approvals, and with a signed data access agreement.
